# Empowering Independence for Visually Impaired Museum Visitors Through Enhanced Accessibility

**DOI:** 10.3390/s25154811

**Published:** 2025-08-05

**Authors:** Theresa Zaher Nasser, Tsvi Kuflik, Alexandra Danial-Saad

**Affiliations:** 1 Department of Information Systems, University of Haifa, Haifa 3498838, Israel; zzaher01@campus.haifa.ac.il; 2 Department of Occupational Therapy, University of Haifa, Haifa 3498838, Israel; asaad@univ.haifa.ac.il

**Keywords:** museum accessibility, blind and partially sighted visitors (BPS), interactive tangible user interfaces (ITUIs), independent exploration, user control

## Abstract

**Highlights:**

**What are the main findings?**

- Enhanced interactive tangible user interfaces (ITUIs) with customizable audio controls significantly improved usability, perceived independence, and user satisfaction among blind and partially sighted (BPS) museum visitors.
- The Pushbutton-based ITUI outperformed Autoplay- and RFID-based systems, achieving the highest ratings in user control (76%) and perceived independence (72%).

**What is the implication of the main finding?**

- Providing BPS users with control over audio playback speed and volume, along with clear tactile feedback, is essential to fostering independent exploration in museums.
- Simple, user-centered interface design may be more effective than complex technologies in supporting accessible and autonomous cultural engagement of BPS visitors.

**Abstract:**

Museums serve as essential cultural centers, yet their mostly visual exhibits restrict access for blind and partially sighted (BPS) individuals. While recent technological advances have started to bridge this gap, many accessibility solutions focus mainly on basic inclusion rather than promoting independent exploration. This research addresses this limitation by creating features that enable visitors’ independence through customizable interaction patterns and self-paced exploration. It improved upon existing interactive tangible user interfaces (ITUIs) by enhancing their audio content and adding more flexible user control options. A mixed-methods approach evaluated the ITUI’s usability, ability to be used independently, and user satisfaction. Quantitative data were gathered using ITUI-specific satisfaction, usability, comparison, and general preference scales, while insights were obtained through notes taken during a think-aloud protocol as participants interacted with the ITUIs, direct observation, and analysis of video recordings of the experiment. The results showed a strong preference for a Pushbutton-based ITUI, which scored highest in usability (M = 87.5), perceived independence (72%), and user control (76%). Participants stressed the importance of tactile interaction, clear feedback, and customizable audio features like volume and playback speed. These findings underscore the vital role of user control and precise feedback in designing accessible museum experiences.

## 1. Introduction

Museums are vital for conserving and displaying human cultural heritage, serving as repositories of artifacts and spaces for learning, inspiration, and cultural engagement [1]. The primarily visual nature of museum exhibits, however, creates significant barriers for blind and partially sighted (BPS) visitors, who number approximately 285 million worldwide [2]. This lack of access poses both a practical challenge and a fundamental issue in cultural inclusion and social fairness [3,4].

Recent technological advancements have opened unprecedented opportunities to improve museum accessibility. Three-dimensional (3D) scanning and printing, interactive audio systems, and tangible user interfaces have become promising tools for creating more inclusive museum experiences [4]. However, as Vaz et al. [5] noted, despite these technological advances, a significant gap remains between providing basic access and enabling truly independent exploration.

When considering the ability of BPS visitors to explore museum exhibits independently, we must consider two aspects: the first is understanding how to navigate the exhibit, and the second is the ability to control the audio commentary. Hence, in this study, independence refers to the user’s ability to control the pace, sequence, and structure of their interaction with exhibits without needing help from mediators or external guidance. Many studies have examined the challenges and barriers that limit BPS visitors’ independence when visiting museums [4,5,6,7,8]. Specifically, a comprehensive survey by Sylaiou and Fidas [6] showed that despite significant technological progress, museums still face major challenges in offering truly independent experiences for BPS visitors. Their analysis of 127 cultural institutions highlights ongoing accessibility barriers that strongly affect visitor independence and engagement. These barriers include conservation-mandated restrictions on tactile exploration, limited spatial and artistic detail in audio descriptions, navigational difficulties within complex museum layouts, inconsistent accessibility standards across institutions, limited integration between physical and digital solutions, and insufficient staff training in supporting BPS visitors. Pirrone et al. [7] took a different approach in another survey. Their systematic literature review also highlights critical gaps in cultural accessibility, emphasizing the need for solutions that address both physical and digital barriers simultaneously. They identified several key issues: the lack of standardized methods for creating accessible exhibits, poor integration between physical and digital accessibility solutions, insufficient metrics for measuring independence in museum experiences, limited personalization options in current solutions, and minimal involvement of BPS users in the design process. Their analysis shows that despite greater institutional recognition of accessibility’s importance, current efforts still fall short of enabling true independent exploration. Similarly, Brischetto et al. [4] emphasized that information related to independent enjoyment by people with sensory disabilities is often lacking, with accessibility typically guaranteed only through pre-scheduled visits and mediators, limiting visitors’ freedom to experience artworks on their terms.

Building upon the work of Avni et al. [9] and recent findings from Brischetto et al. [4], this research investigates how ITUIs can empower BPS visitors to engage with museum exhibits independently. Early ITUI prototypes combined 3D-printed replicas with audio descriptions through different interaction methods and provided valuable insights into user preferences and interaction patterns while highlighting key areas where enhanced functionality could increase user independence. Our research extends the work of Avni et al. [9] by focusing on three ITUI prototypes—Autoplay, Pushbutton, and radio-frequency identification (RFID) scanning (using an RC522 RFID 13.56 MHz Reader Writer Module)—and improving their audio and users’ control with tactile feedback can improve the independent exploration capabilities of BPS visitors. Our study extends previous work by systematically comparing interaction paradigms through quantitative measures of user independence, moving beyond qualitative insights alone.

The research question guiding this work is as follows: How do enhanced tactile, customized audio description and audio control features integrated into ITUIs affect the exploration of independent museum exhibits for BPS visitors?

In seeking to resolve our research question, we offer two hypotheses:

**H1:**  *Interfaces offering higher user control, especially customized audio playback speed and volume, will increase perceived independence and usability among BPS visitors compared to systems with fixed or limited controls.*

**H2:**  *Tactile clarity, combined with enhanced audio feedback and customization, will positively correlate with user satisfaction and independence ratings, thus promoting autonomous cultural exploration among BPS visitors.*

This study employed a mixed-methods approach involving 25 BPS participants who evaluated these enhancements. Data were collected through usability questionnaires, satisfaction ratings, and comparative assessments. Qualitative data were collected from think-aloud protocols, direct observation, and video analysis. The results confirmed our hypotheses about the effect of audio control on the sense of independence.

This study contributes to the field by providing an evidence-based model for developing accessible museum technologies that support autonomous cultural engagement, with practical implications for ITUI design and implementation in cultural institutions.

## 2. Background and Related Work

Over the years, the potential of state-of-the-art information and communication technologies to enhance the museum experience for BPS visitors has grown. The literature identifies three principal strategies for enhancing accessibility: (1) standalone tactile replicas, (2) integrated multimodal systems, and (3) interactive user-controlled systems [10,11]. This section examines the current state of accessibility technologies in museums and related research, with particular focus on interactive tangible user interfaces (ITUIs) and their role in promoting independent exploration for BPS visitors, and it then reviews recent development frameworks and evaluation methodologies.

### 2.1. Museum Accessibility Technologies

#### 2.1.1. Standalone Tactile Replicas

The most basic approach, standalone tactile replicas, is widely adopted across cultural institutions. For example, De-Miguel-Sánchez and Gutiérrez-Pérez [12] reported that 80% of 15 European museums surveyed offer 3D-printed models, yet only 35% incorporate these with synchronized audio descriptions. This is consistent with findings by Papis et al. [13], who emphasized that while 3D-printed reproductions enhance physical engagement, their effectiveness in conveying meaning is limited without complementary auditory or textual information. Similarly, broader critiques in the literature suggest that although tactile models provide valuable sensory access, the absence of interpretive guidance hinders the user’s ability to grasp spatial relationships and contextual significance [14,15,16]. While tactile models provide valuable sensory access, their impact remains constrained without interpretive guidance.

#### 2.1.2. Integrated Multimodal Systems

Integrated multimodal systems have shown greater promise by combining tactile artifacts with audio or haptic feedback. Montusiewicz et al. [17], for example, developed a location-aware audio guide paired with 3D-printed replicas at the Archaeological Museum of Kraków. Their implementation significantly improves BPS visitors’ engagement and spatial understanding. Similarly, Karaduman et al. [11] combine scaled replicas and touch-triggered audio in their “Touch and Learn” initiative, embedding historical narratives into tactile interaction. Shi et al. [18] introduced Molder, a tool for co-designing tactile maps for blind users, and [19] collaborated with teachers of the visually impaired to explore interactive 3D models with embedded feedback. These efforts illustrate the growing recognition that multimodal presentation, not merely tactile access, is critical for meaningful engagement. These systems employ innovative materials and responsive interfaces to enhance tactile and auditory feedback.

Successful systems combine tactile exploration with layered contextual information, integrate multisensory feedback, and comply with universal design standards [10,17,20]. Reinders et al. [21] explored the embodied experience of multimodal 3D models, emphasizing how touch, sound, and interaction merge to “bring the model to life”. In a broader review, Jiang et al. [22] analyzed the role of haptic feedback in BPS user interfaces. Additionally, Rector et al. [23] investigated audio hierarchy control in art engagement, showing how proxemic audio delivery allows users to guide the flow of layered information, an approach relevant to the structure of audio used in this study. Many installations, however, still rely on fixed narrative pacing, limiting the user’s control and undermining autonomy, an issue repeatedly flagged in recent reviews [4,5].

#### 2.1.3. Interactive, User-Controlled Systems

The persistence of limited interactivity across systems reveals a critical design shortfall: a lack of sufficient user agency. Research emphasizes that flexible features, such as adjustable playback, repeatable content, and customizable exploration paths, are essential for fostering independence [5,8]. Interactive systems incorporating audio descriptions have become essential in shaping accessible museum experiences for BPS visitors.

### 2.2. Interactive Tangible User Interfaces (ITUIs)

ITUIs have emerged as a promising solution for combining physical accessibility with enhanced user control. For instance, Leporini et al. [10] propose four core guidelines for effective interactive systems: consistent feedback, user-controlled pacing, multimodal delivery, and adaptable interfaces. These principles are echoed in Wang et al.’s [24] findings, which show that visitor engagement increases when users can control the pace and sequence of audio content. Complementary research by Hutchinson and Eardley [25] demonstrates that well-designed audio descriptions can enhance both cognitive retention and emotional engagement with art, not only for BPS visitors but also for sighted audiences. Expanding this perspective, Wang [8] advocated for descriptive systems that allow personalized interaction, highlighting the importance of individual control in creating meaningful museum experiences and emphasizing how user control over pacing, sequencing, and repetition fosters more meaningful and empowering museum experiences.

Avni et al. [9] sought to determine the correct balance through evaluation of three interactive tangible user interfaces (ITUIs): Autoplay, Pushbutton, and RFID scanning, each of which offers a different level of user control. Their findings emphasize the value of clear and accessible audio description, tactile clarity, and interaction flexibility, aligning closely with the design principles laid out by Leporini et al. [10] and others. Similary, Cavazos Quero et al. [26] developed an interactive multimodal guide combining haptic, audio, and gestural inputs to enhance art accessibility. Their system underscores the need for layered interaction methods that adapt to user preferences and limitations. On a design principle level, Horton et al. [27] synthesized key recommendations for tactile technology, stressing low physical effort, feedback consistency, and error tolerance—principles that underpin effective ITUI implementation. Furthermore, Jiang et al. [22] provided a comprehensive review of haptic feedback systems for BPS users, emphasizing how combining tactile signals with audio enhances spatial awareness, supporting the multimodal approach adopted in this study.

Critical design considerations for ITUIs include material selection, scale design, and tactile quality optimization [12]. Montusiewicz et al. [17] provided empirical evidence for specific technical thresholds, such as texture variation ranges and durability standards, affirming that well-executed tactile models significantly improve object recognition and comprehension.

### 2.3. Development Considerations and Frameworks for User Independence and Control

Among the existing frameworks that have emerged to define how tactile technologies, interaction methods, and content delivery systems can be optimized to promote user independence, De-Miguel-Sánchez and Gutiérrez-Pérez [12] made a foundational contribution. They presented a structured methodology for creating accessible cultural heritage experiences through 3D printing. Their approach identifies three core domains: material selection, which must optimize durability and tactile sensitivity; scale design, which should account for ergonomic handling and preservation of meaningful detail; and tactile quality, which involves refining surface texture gradients and structural integrity to enhance perceptibility. These principles lay the groundwork for producing tactile models that are both informative and intuitive to explore. Building on these foundations, Montusiewicz et al. [17] contributed empirical evidence acquired through extensive user testing that lays down specific technical thresholds, such as texture variation ranges and durability standards. Their findings affirm that well-executed tactile models significantly improve object recognition and comprehension, reinforcing the value of precise, user-centered design at the physical level. Still, physical accuracy alone does not ensure independent use. In addition to the tactile qualities of replicas, the way users interact with the system—and how the system responds—is equally important. Shehade and Stylianou-Lambert [28] stressed the importance of balancing technological innovation with practical usability. While advanced features such as presence-detection technologies may enrich the experience, they caution that overly complex systems can overwhelm users and undermine their autonomy. Similarly, Vaz et al. [5] highlighted that interaction methods must support intuitive navigation and allow users to engage with content at their own pace. Systems that are too rigid or lack clear feedback can hinder rather than help independent exploration. Another critical aspect is audio accessibility, which plays a central role in the museum experience for BPS visitors. Snyder [20] and Wang [8] emphasized that audio description systems should offer precise, user-driven control such as the ability to pause, repeat, adjust volume, or change playback speed. These features are essential for enabling visitors to absorb complex information in a way that matches their individual needs and learning pace, supporting both comprehension and confidence.

Ballarin et al. [29] discussed the role of 3D-printed replicas in shaping museum experiences, emphasizing their contribution to both physical accessibility and public engagement; however, they caution that replicas must be meaningfully integrated into narrative structures to avoid becoming mere novelties. In the same vein, Ludovico and Mario [30] explored how digital fabrication techniques can facilitate inclusive cultural practices, particularly when co-designed with disabled audiences. Their work reinforces the value of participatory design in developing tactile museum content.

In addition to institutional and design-related barriers, Theodorou and Meliones [31] highlighted the value of remote and user-centered methods in designing assistive systems for visually impaired users, emphasizing that flexible testing environments can yield reliable insights while prioritizing user comfort. This approach aligns with recent methodological shifts in accessibility research, prioritizing user comfort and flexibility. Complementing this, Comes [32] further elaborated on the potential of haptic interfaces in museums, describing how tactile experiences can bridge the sensory gap for BPS visitors by providing an alternative mode of engagement beyond audio descriptions. Moreover, Dimitrova-Radojichikj [33] stressed that physical access alone does not guarantee meaningful participation for visitors with visual impairment, pointing to persistent structural and educational barriers in exhibition planning and staff training. Similarly, Mesquita and Carneiro [34] argued that museum communication should be inclusive and dialogic, advocating for adaptive interpretive approaches that accommodate diverse sensory and cognitive needs—an important perspective when considering user autonomy and content delivery methods.

From a policy standpoint, Landau et al. [35] outlined early frameworks and standards for museum accessibility for BPS individuals. Their work underscores foundational gaps that modern implementations still seek to address, particularly concerning tactile guidance and user-centered content. In another study, Wang [8] validated these findings, emphasizing that effective accessibility solutions require careful consideration of both technological capabilities and user experience design principles.

### 2.4. Evaluation of Design Frameworks

Given the variety of development frameworks and guidelines, the issue thus becomes how to evaluate the effectiveness of them and their products. Bevan [36] suggested using international usability standards in HCI, offering foundational benchmarks to assess ITUI performance regarding usability, efficiency, and satisfaction. Regarding methodological design, Nielsen [37] and Nielsen and Landauer [38] suggested that small user samples can yield reliable usability insights, a concept particularly relevant in accessibility contexts with limited participant pools. From a design perspective, Vredenburg et al. [39] and Ulrich and Eppinger [40] emphasized the value of iterative, user-centered approaches incorporating stakeholder feedback—principles that influenced the development of the ITUIs examined in this study. Complementing this, Black [41] argued for participatory engagement in museums, reinforcing the importance of designing systems that empower users rather than prescribe passive interaction.

### 2.5. Existing Challenges

Despite technological advances, significant gaps persist in museum accessibility research. Comprehensive surveys [6,7] identify persistent barriers including inadequate spatial detail in audio descriptions, limited integration between physical and digital solutions, and critically, insufficient metrics for quantifying independence in museum experiences.

Current research lacks a systematic comparison of interaction paradigms using quantitative measures of user independence. While qualitative insights have informed design principles, evidence-based models for developing accessible museum technologies remain limited. Furthermore, the relationship between specific ITUI features (such as enhanced audio control and tactile feedback) and measurable independence outcomes has not been systematically investigated.

Building upon Avni et al.’s [9] foundational work, this study addresses these gaps by (1) systematically comparing ITUI interaction paradigms, (2) employing quantitative measures of user independence, and (3) investigating the relationship between enhanced features and independence outcomes. This approach extends existing theoretical frameworks by providing empirical evidence for design decisions that support autonomous cultural engagement for BPS visitors.

## 3. Materials and Methods

### 3.1. Participants

A total of 25 BPS participants (15 female, 10 male, age range 19–58 years, M = 31.2, SD = 11.4) were recruited through partnerships with the Hand in Hand for Blind People Association (*n* = 10) and AlManarah Association (*n* = 15). Sample size determination was based on statistical Gpower (version 3.1.9.7) considerations for within-subjects comparison across three prototype ITUIs (Autoplay, Pushbutton, and RFID scanning). Using standard parameters (α = 0.05, power = 0.80) and assuming a medium effect size (f = 0.25) based on Cohen’s [42] guidelines for behavioral research, preliminary power calculations performed in SPSS version 27.0 indicated a minimum required sample size of 23 participants. The final sample size of 25 participants was selected to account for potential attrition while maintaining sufficient statistical power for the planned analyses.

Inclusion criteria required participants to be blind or severely partially sighted and aged 18 or older. Participants demonstrated sufficient cognitive ability for informed consent and had no additional disabilities that might impact ITUI interaction (detailed demographic information is presented in Appendix A).

Exclusion criteria were individuals with additional disabilities that could impact ITUI interaction, those unable to commit to the complete testing session, and those with cognitive or language barriers that might affect task comprehension.

### 3.2. Experimental Prototypes

In this section, we provide first the description of our original prototypes and then explain the modifications made for enabling independent experience of BPS visitors.

#### 3.2.1. The First Prototype

**Figure 1:**
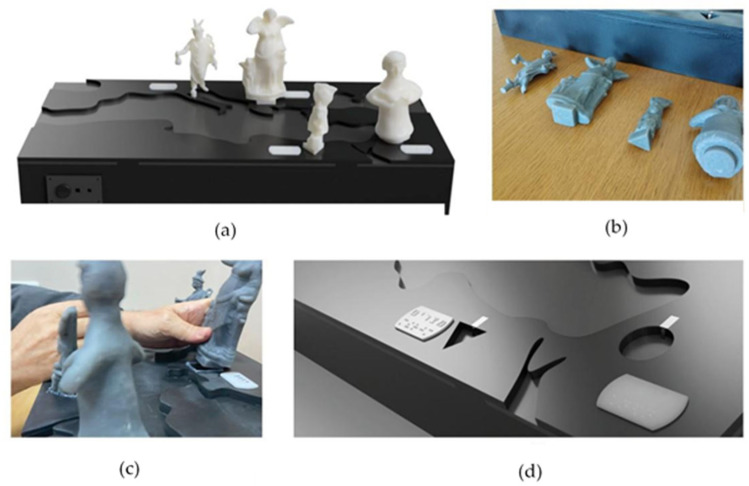
The Mythology and Autoplay ITUI: (**a**) The item is placed where it was initially found in the Mediterranean basin. (**b**) Each item has a similar shape embossed on the bottom, with matching recesses on the top surface of the ITUI. (**c**) Audio files initiate when a replica is removed from its place. (**d**) Three-dimensional tag with the origin country name in Hebrew and Braille next to each item’s place.

The prototype, Mythology (Figure 1), utilizes an Autoplay interaction model and features four standing replicas representing deities from different Mediterranean cultures: Mercurius (Rome), Nike (Greece), Ashera (Canaan), and Isis (Egypt). This prototype represents a thematic exploration of Mediterranean mythology, aiming to convey cultural diversity and shared symbolism across ancient civilizations. The selected deities were chosen for their strong visual and narrative identities, and each reflects the religious beliefs, societal roles, and regional aesthetics of its origin’s culture. The geographical layout of the replicas reinforces historical spatial understanding, allowing users to tangibly grasp both the individuality and interconnectedness of Mediterranean mythologies, as curated in the Hecht Museum’s collection [9]. The ITUI is designed in the shape of a map of the Mediterranean basin, with each replica positioned according to its geographic origin. Each replica is placed into a uniquely shaped socket, enabling tactile guidance for orientation and accurate placement. When a user removes a replica from its socket, an audio description automatically begins playing, providing detailed information about the figure. Returning the replica to its socket stops the playback. Each socket is embedded with a microswitch that detects the presence or absence of the replica, which serves as the activation mechanism for the audio output. Braille and printed labels next to each figure support additional identification. A brief introductory explanation is triggered when the ITUI is powered on, a feature consistent across all three prototypes presented in this study. This Autoplay mechanism, which links physical interaction to automatic audio feedback, is designed to support effortless, hands-free engagement while maintaining a clear correspondence between tactile exploration and information delivery.

#### 3.2.2. The Second Prototype

**Figure 2:**
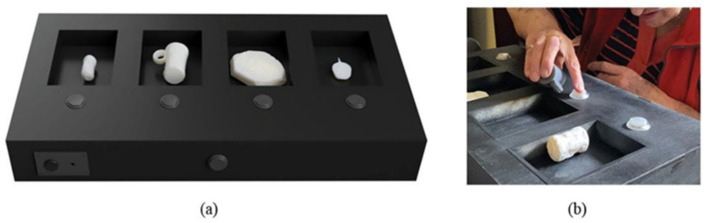
The Writing and Pushbuttons ITUI: (**a**) The ITUI comprises four items: (right to left) charm, legion tile, inkwell, and cylinder seal. (**b**) A Pushbutton is positioned in front of every box corresponding to the piece.

The second prototype, Writing (Figure 2), comprises four replicas: a charm, a legion tile, an inkwell, and a cylinder seal. The Writing ITUI centers around the theme of communication and symbolic expression in antiquity. Each replica exemplifies a distinct form of writing or inscription used in the ancient Near East and the Greco-Roman world. The cylinder seal, for instance, served as a form of identity authentication in Mesopotamian societies, while the Roman legion tile contains official inscriptions. This prototype encourages comparative reflection on the evolution of writing technologies and their societal implications, reflecting the museum’s emphasis on literacy and administration in ancient times [9]. It utilizes a Pushbutton to activate audio files. Each replica is placed in a compartment, with a Pushbutton positioned in front of the compartment corresponding to the replica. Additionally, a Pushbutton located on the middle front wall initiates an introduction to the main topic of the ITUI (Figure 2a). Users can take a replica from a compartment and press the button to play or stop the audio file (Figure 2b). The order in which the replica is presented has no meaning for the overall understanding, meaning the user can use it in any order. This prototype was designed in response to pilot feedback from visually impaired users who emphasized the value of direct control over audio content. The Pushbutton interface allows users to decide when to initiate or stop playback, offering a sense of agency absent in passive systems. Each button is strategically aligned with its corresponding replica compartment to promote spatial consistency and reduce cognitive load, aligning with best practices in assistive interface design noted by Avni et al. [9]. A power toggle switch is situated on the front left wall of the ITUI to manage the power ON or OFF. When the power is ON, an overall explanation of the ITUI is automatically played.

#### 3.2.3. The Third Prototype

**Figure 3:**
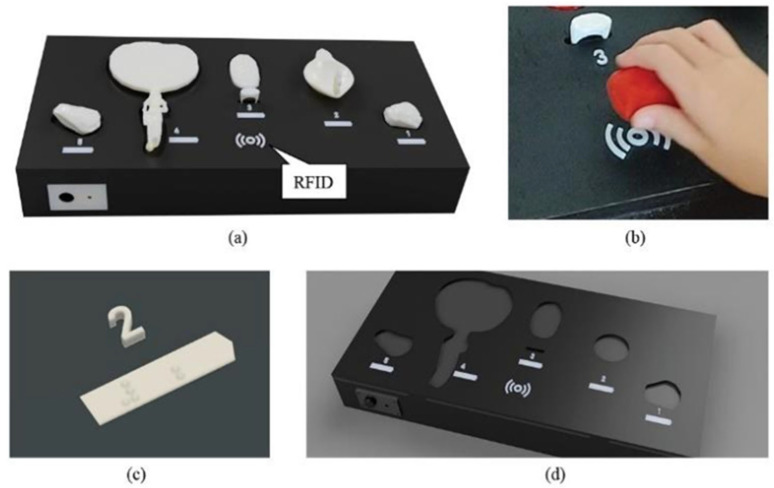
The Burial Tradition (RFID scanning), linear order ITUI: (**a**) Items arranged in linear order. (**b**) Audio is played by scanning the replicas. (**c**) Both digits and Braille numbers are used. (**d**) Each item has fixed recesses placed on the top surface.

In the third prototype, Burial Tradition (Figure 3), RFID technology activates audio files through scanning [43]. An RFID tag is positioned at the bottom of each replica, with a textured surface placed atop the tags to assist users in locating them. The RFID-based interaction was conceptualized as a metaphor for archaeological exploration, where information is “unearthed” through scanning. This model fosters an investigative experience, encouraging users to actively seek knowledge. The linear spatial arrangement, complemented by both embossed digits and Braille numbering, supports narrative coherence and orientation, making it easier for users to progress through the content in a logical, memorable sequence. These features were inspired by prior research on sequential tangible interaction models, as explored by Avni et al. [9].

To listen to an explanation about a specific replica, users position the item approximately 1–2 cm away from the scanning icon situated in the middle front of the ITUI (Figure 3a). The audio file will play either until its conclusion or until the same tag is scanned again. A power toggle switch (Figure 3b) is located on the front left wall of the ITUI to control the power ON or OFF. When the power is ON, an overall explanation of the ITUI is automatically played. The ITUI comprises five items arranged in linear order from right to left (Figure 3a), including (1) the introduction to the ITUI, (2) an alabaster duck, (3) a seal ring and a scaled detail of the seal ring top, (4) a bronze mirror, and (5) a summary. The Burial Tradition ITUI introduces objects associated with funerary practices and personal belongings placed in tombs, reflecting beliefs about death, identity, and the afterlife in the ancient Levant. The alabaster duck, for instance, was commonly used as a symbolic grave good, while the bronze mirror and seal ring represent personal items tied to social status and remembrance. By presenting these artifacts in a linear narrative, the ITUI guides users through a conceptual journey of burial customs, echoing interpretive strategies used in museum exhibitions to foster emotional and cultural connections (Avni, [9]). Each item is numbered with both embossed digits and Braille numbers (Figure 3c). Braille labels were created using TouchSee.me, a free online tool that generates standard-compliant 3D-printable models and STL files (files suitable for 3D printers) of Braille labels. Each item has a fixed place on the top of the ITUI, having the same shape as the object placed in it to guide users in returning it to its proper place (Figure 3c).

### 3.3. Enhancements

Our focus was on refining three existing ITUI prototypes: Autoplay, Pushbutton, and RFID scanning, to overcome the barriers to independent interaction—insufficient tactile feedback, lack of spatial orientation cues, and limited user control, among others, identified by Avni et al. [9]. The design modifications were guided by user-centered principles and shaped by prior feedback from BPS participants, which emphasized the importance of intuitive navigation, detailed content delivery, and flexible control mechanisms. The enhancements focused on two core aspects: restructuring audio descriptions and expanding user control through adjustable audio volume and playback speed. Audio description protocols for BPS individuals emphasize the need to convey visual information and provide clear orientation cues [20]. Building on these principles, the revised audio descriptions integrate three key components: spatial context that situates each exhibit on the ITUI surface, detailed hand navigation instructions, and precise handling guidance describing the replicas’ physical characteristics and optimal interaction strategies. Structuring the descriptions hierarchically—from overview information to detailed content—addresses a critical barrier to independent exploration identified in previous studies [44].

Following the Smithsonian Institution’s guidelines [45], each description adheres to a consistent pattern comprising orientation information, physical characteristics, historical context, and usage guidance. This systematic approach supports users in constructing accurate mental models of the objects and their surrounding space, thereby enhancing independent navigation [46].

**Figure 4:**
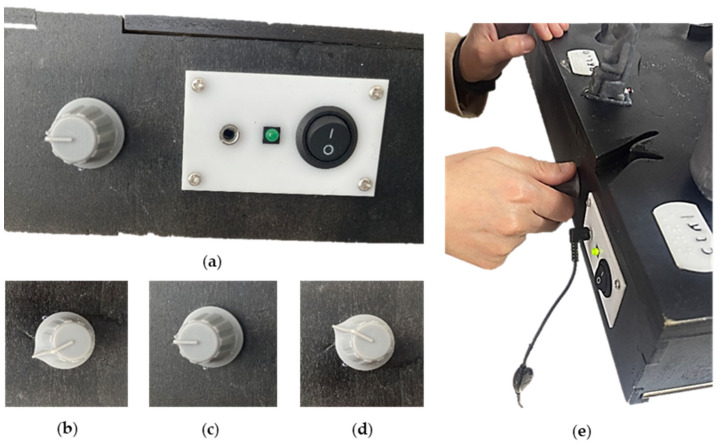
Playback speed control module. (**a**) The playback speed control module is next to the headphones plug and the ON/OFF switch. (**b**) The playback speed control module pointing to the lowest speed ×0.8. (**c**) The playback speed control module pointing to the normal speed ×1.0. (**d**) The playback speed control module pointing to the highest speed ×1.5. (**e**) One of the participants using the playback speed control module during the experiment.

**Figure 5:**
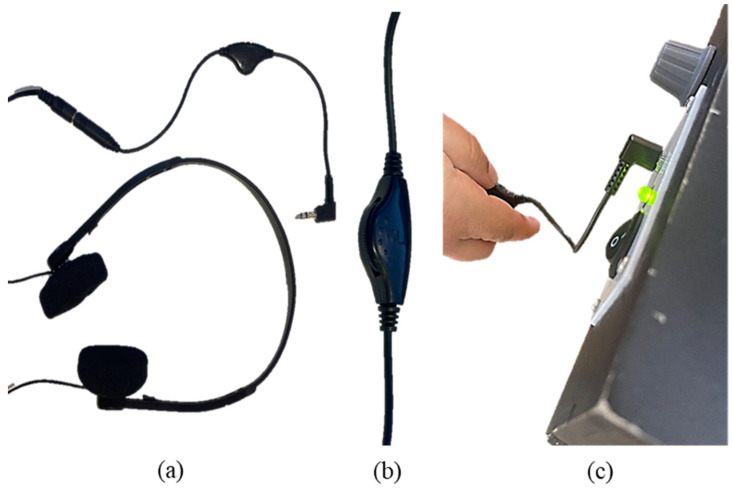
Volume control module. (**a**) The volume control module is connected to the headphones. (**b**) The volume control pulley. (**c**) One of the participants using the volume control module during the experiment.

User control over the audio experience represents another major enhancement. Prior research highlights that BPS users often require variable processing times for audio content, particularly when engaged in simultaneous tactile exploration. To meet these needs, variable playback speed functionality was incorporated, as shown in Figure 4, allowing users to adjust the pace of audio content according to their preferences. Similarly, volume control capabilities were introduced, as illustrated in Figure 5, to accommodate diverse acoustic environments and individual hearing needs, in line with findings from Bandukda et al. [47] and Gleason et al. [48].

These improvements were systematically integrated across the three interaction paradigms evaluated in this study. In the Autoplay ITUI, automatic audio activation triggered by object manipulation is enhanced by the addition of volume and playback speed controls, thereby maintaining accessibility while expanding user agency, consistent with principles outlined by Reichinger et al. [49]. The Pushbutton ITUI incorporates tactilely distinct controls with ergonomic placement, facilitating intuitive operation while enabling users to manage audio features independently, under design guidelines proposed by Horton et al. [27]. In the RFID scanning ITUI, contactless activation is supplemented with enhanced feedback mechanisms, along with volume and playback speed controllers, building upon the interaction framework established by D’Agnano et al. [50].

These integrated modifications collectively reflect a user-centered design approach aimed at enhancing autonomy, improving usability, and fostering a more inclusive museum experience for blind and partially sighted visitors.

### 3.4. Measures

Usability was assessed using the System Usability Scale (SUS) [51], a ten-item instrument widely used in human–computer interaction research. Each ITUI was rated separately on a five-point Likert scale. Scores were converted to a 0–100 composite usability index. Participant satisfaction was measured using a modified version of the Quebec User Evaluation of Satisfaction with Assistive Technology (QUEST 2.0) [52], extended with seven additional user satisfaction questions (AUSQ) developed specifically for this study. These AUSQ items targeted tactile responsiveness, control reliability, perceived safety, and physical comfort and were rated on a five-point scale. To explore preferences, a two-part questionnaire (TPQ) was administered. TPQ1 assessed six dimensions—ease of use, comfort, efficiency, safety, learning, and overall preference—on a five-point Likert scale. TPQ2 measured future expectations regarding museum accessibility via nine items rated on a seven-point Likert scale. Item phrasing was informed by validated usability and universal design instruments. Examples include: “The ability to change the reading speed of the audio system is important” and “The ability to touch and hold the printed exhibits enhances understanding” [4,49]. Qualitative data were collected through think-aloud protocols [53], open-ended comment sections, and video-recorded sessions. Participants verbalized their thoughts during interaction, and these verbalizations were transcribed and coded. Comments were thematically analyzed using Braun and Clarke’s six-phase reflexive thematic analysis framework [54]. Two independent coders developed codes iteratively, focusing on emergent themes such as spatial orientation, control granularity, and user feedback. Discrepancies were resolved by consensus. All sessions were video-recorded to capture non-verbal behavior, including hesitation, repeated gestures, and scanning movements. These recordings were analyzed using structured behavioral observation methods [55,56], focusing on interaction challenges such as tactile misalignment or repeated failed attempts. This observational method aligns with established usability testing practices that incorporate behavioral data to complement quantitative and qualitative feedback [57]. A structured coding scheme included categories like “interaction delay,” “need for guidance,” and “re-engagement,” providing a complementary behavioral layer to the self-report measures.

### 3.5. Procedure

To evaluate the effectiveness of the enhancements, this study employed a mixed-methods approach. The research protocol received approval from the Ethics Committee for Human Experiments at the University of Haifa (IRB approval number 078/24). All participants provided written informed consent.

To ensure accessibility and ecological validity, testing was conducted in familiar and comfortable environments—either at participants’ homes (*n* = 15) or at partner organization offices (*n* = 10) [15,58]. Each site was set up with three stations, one for each ITUI. Prototype presentation order was counterbalanced using a Latin square design [59], with participants randomly assigned to one of six sequences. A standardized checklist [60,61] was used to ensure consistency across locations, covering equipment positioning, functionality checks, video device calibration, and spatial arrangements. Recordings were captured via an iPhone or iPad positioned overhead, following protocols from assistive technology usability studies [55,56].

Each session lasted approximately 60 min and followed a structured workflow. The first 10 min involved informed consent, an explanation of procedures, and administration of the demographic questionnaire. Then, participants completed three ITUI evaluation cycles. Each cycle included a brief training phase (~5 min), an independent interaction phase (4–7 min) under the think-aloud protocol, and an evaluation phase (4–6 min), during which participants completed the SUS. In the final stage of the session, participants completed the AUSQ and TPQ instruments and provided open-ended feedback. This multi-phase process ensured systematic data collection while allowing for flexibility based on participant needs and comfort.

### 3.6. Data Analysis

Quantitative data were analyzed using SPSS version 27.0. Repeated-measures ANOVA tested for differences between the three ITUIs (Autoplay, Pushbutton, RFID). Where significant main effects were found, Tukey’s Honestly Significant Difference (HSD) tests were used for post hoc pairwise comparisons. Pearson’s correlation coefficients were calculated to assess associations between participants’ satisfaction with specific user control features (e.g., volume adjustment, playback speed control) and perceived independence scores. Chi-square tests were used to compare preference distributions across ITUIs. Effect sizes were calculated using Cohen’s d, with *p*-values < 0.05 considered statistically significant [62].

Complementing the quantitative data, qualitative insights were derived through inductive thematic analysis of open-ended questionnaire responses, think-aloud comments, video recordings, and researcher field notes. This approach followed the six-phase framework proposed by Braun and Clarke [54], allowing for the identification of recurring perceptions related to ease of use, control, engagement, accessibility, and perceived independence. Coding was conducted iteratively, beginning with the generation of initial codes, grouping them into candidate themes, and refining them to capture both individual variations and cross-participant patterns.

Video recordings were analyzed using structured behavioral observation techniques [55,56], focusing on non-verbal interaction cues such as hesitation, exploratory hand movements, repeated gestures, and instances of re-engagement with the ITUI interfaces. These behavioral markers were triangulated with user-reported data to reveal usability issues not always evident through self-report measures alone. Video analysis focused on identifying non-verbal indicators of usability challenges, such as hesitation, scanning movements, repeated attempts to activate interfaces, or verbal cues of confusion. A structured coding scheme was developed based on established assistive technology observation protocols (e.g., [55]) and included categories such as “interaction delay,” “tactile misalignment,” “need for guidance,” and “repeated interaction.” These codes were applied to each participant session by two independent raters, with disagreements resolved through consensus discussion to enhance reliability. 

Together, the combined quantitative and qualitative analyses provide a holistic assessment of each ITUI’s effectiveness.

## 4. Results

This section presents the findings from the evaluation of the three ITUI prototypes. It is organized into three main parts: (1) participants’ museum visit behavior and baseline experiences, (2) quantitative results based on usability and satisfaction metrics, and (3) qualitative findings derived from think-aloud protocols, video observations, and open-ended feedback. Together, these results provide a comprehensive picture of how interaction design influences independent exploration and user satisfaction among BPS visitors.

### 4.1. Descriptive Findings: Museum Visit Behavior

**Figure 6:**
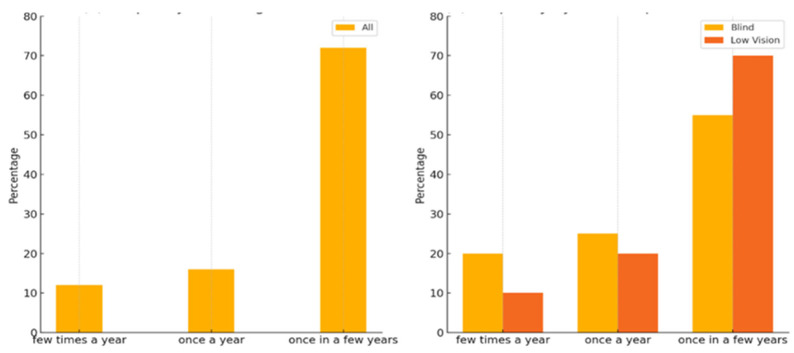
Visiting museums: (**a**) Frequency of visiting museums. (**b**) Frequency by visual impairment definition.

Regarding museum experience, most participants reported infrequent museum visits regardless of their visual impairment definition (Figure 6a). Prominently, approximately 60% of respondents indicated that they visit a museum “once in a few years,” with fewer participants reporting visiting museums “once a year” or “a few times a year.” This trend highlights a significant gap in regular cultural engagement among individuals with visual impairments, suggesting that accessibility barriers discourage frequent participation in museum activities. Among those who visited museums, 91% relied on guided tours or family assistance, highlighting the current dependence on external support for museum experiences (Appendix A). Further examination of the data reveals notable differences in museum visit frequencies based on the definition of visual impairment (Figure 6b). Participants categorized as “blind” demonstrated a slightly higher propensity for occasional visits compared to those defined as “visually impaired” (low vision). Among blind participants, nearly 20% reported visiting museums “once a year” or “a few times a year.” In contrast, visually impaired participants predominantly reported infrequent visits, with nearly 70% indicating that they visit museums only “once in a few years.” 

### 4.2. Quantitative Analysis

**Figure 7:**
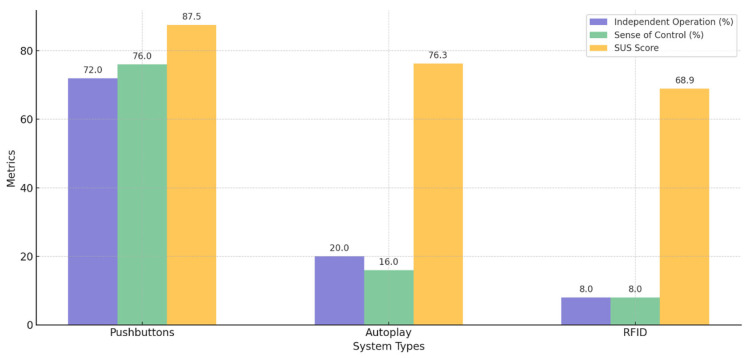
Independence and control analysis according to the interaction methods.

The analysis of the usability and independence metrics revealed significant differences in perceived autonomy and control capability across the three ITUI prototypes. The Pushbutton interface was preferred over the other two interfaces (Autoplay and RFID interfaces) across most dimensions. Figure 7 summarizes the results of the different metrics.

As Figure 7 and Table 1 (below) show, the Pushbutton ITUI achieved the highest overall SUS score (M = 87.5, SD = ±8.2; see Appendix D for details), significantly exceeding both the Autoplay ITUI (M = 76.3, SD = ±9.4) and the RFID ITUI (M = 68.9, SD = ±11.2). (In Table 1, values include mean and standard deviation (SD) for continuous measures and frequency (%) for categorical measures.) A repeated-measures ANOVA confirmed a significant main effect of ITUI type on usability scores, F(2, 72) = 12.34, *p* < 0.001, η^2^ = 0.26.

To determine the source of these differences, Tukey’s HSD post hoc analysis was conducted. The results showed that the Pushbutton ITUI scored significantly higher than both the Autoplay (*p* = 0.011) and RFID scanning (*p* < 0.001) interfaces. The difference between Autoplay and RFID was not statistically significant (*p* = 0.087).

Perceived independence followed a similar pattern. The majority of the participants, 72% (*n* = 18), rated the Pushbutton ITUI as the most independent, followed by the Autoplay ITUI (20%, *n* = 5) and the RFID ITUI (8%, *n* = 2). These differences were statistically significant (χ^2^(2) = 19.28, *p* < 0.001). Similarly, for sense of control, the Pushbutton ITUI was rated highest by 76% of participants (*n* = 19), with the Autoplay ITUI at 16% (*n* = 4) and RFID ITUI at 8% (*n* = 2), showing significant preference differences (χ^2^(2) = 21.44, *p* < 0.001).

We note that although the study included 25 participants, the ANOVA degrees of freedom (e.g., F(2, 72)) reflect a within-subjects repeated-measures design, in which each participant provided ratings across all three interface conditions (Pushbutton, RFID, and Autoplay). This structure results in multiple observations per participant, justifying the reported degrees of freedom.

Further support for these findings was observed in the general preferences questionnaire (Appendix B.3). Tactile interaction was rated highly important for understanding (M = 6.4, SD = ±0.8), with 88% of participants rating it 6 or 7 on the seven-point scale. Audio descriptions significantly contributed to independent understanding (M = 6.2, SD = ±0.9), with 84% giving high ratings. Statistical analysis revealed a strong positive correlation between control feature satisfaction and overall independence ratings (r = 0.78, *p* < 0.001), indicating that the effective implementation of control features correlates with users’ sense of autonomy.

**Figure 8:**
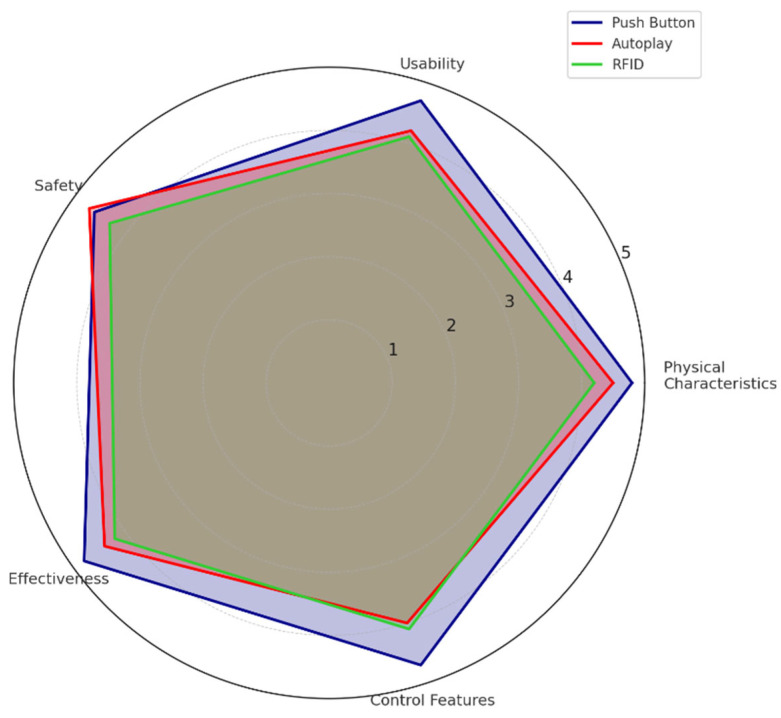
User satisfaction dimensions by interaction technique (blue = Pushutton, red = Autoplay, green = RFID tag scanning). Values shown are mean ratings on a five-point Likert scale (1 = strongly disagree, 5 = strongly agree).

The results show that the Pushbutton ITUI consistently had high performance across most dimensions, particularly in physical characteristics (4.8/5.0) and effectiveness (4.8/5.0) (as illustrated by Figure 8). The Autoplay ITUI showed comparable performance in safety (4.7/5.0) but lagged in control features (4.0/5.0). It is important to note that even though the differences in the perception of safety are significant, they are minimal between Pushbutton and Autoplay. Table 2 provides the means, standard deviation, and a detailed statistical analysis of these differences, confirming their significance across all dimensions (*p* < 0.05).

Tukey’s post hoc comparisons revealed that the Pushbutton ITUI was rated significantly higher than RFID scanning on all five dimensions (*p* < 0.05) and higher than Autoplay in usability and control (*p* < 0.05). There was no significant difference between Autoplay and RFID in physical characteristics and safety.

### 4.3. Qualitative Feedback Analysis

The analysis revealed three primary factors that significantly influence the effectiveness of the ITUIs: feedback clarity, control granularity, and spatial organization.

Feedback clarity refers to the immediacy, consistency, and unambiguity of system responses following user actions. It emerged as a dominant theme, cited by 88% of participants as critical for confident and independent interaction. Participants highlighted that clear tactile–auditory feedback allowed them to understand quickly whether an action had been successfully executed. For instance, one participant stated, *“When I pressed the button, I immediately knew it worked; it talked back clearly and directly,*” referring to the Pushbutton ITUI. In contrast, another participant described the RFID ITUI as “*confusing and slow to respond — sometimes I didn’t know if it even scanned the object.*” These insights underline the importance of immediate and reliable feedback, particularly for non-visual users relying primarily on touch and hearing to navigate and interact.

Control granularity, defined as the degree to which users can manage, pace, and repeat content autonomously, was emphasized by 84% of participants as essential for fostering independent exploration. Participants praised the Pushbutton ITUI for allowing discrete control over audio playback, enabling them to pause, repeat, and regulate the information flow according to their individual processing needs. One participant noted, *“I loved that I could replay the explanation whenever I missed something—it made me feel like I was in control, not just following along blindly*.” In contrast, the fixed automatic playback of the Autoplay ITUI was criticized for limiting flexibility, with participants expressing frustration at being unable to slow down or revisit information at will.

Spatial organization refers to the physical layout, tactile navigability, and logical arrangement of interaction elements. This theme was identified by 76% of participants as a major factor impacting navigation and cognitive mapping. Participants appreciated the systematic and intuitive arrangement of the Pushbutton ITUI, which facilitated easier learning and spatial orientation. One participant explained, *"It was like a small map in my hands—everything made sense and was where I expected it to be."* Conversely, the less structured scanning zones in the RFID ITUI often caused confusion, with one participant remarking, *“I kept searching for the scanning spot and it was frustrating; I never felt sure where to place the object."*

Across the qualitative data, 19 participants expressed positive views about the Pushbutton ITUI, praising its intuitive layout, responsive feedback, and tactile clarity. Only two participants had critical remarks, primarily about initial confusion with button placement. In contrast, the Autoplay ITUI received mixed responses: five participants viewed it favorably because of its simplicity, while thirteen noted frustration with its lack of control features. The RFID ITUI received the most negative feedback, with 18 participants reporting difficulty locating the scanning zone and uncertainty about whether an object was successfully scanned. Notably, several participants (*n* = 6) mentioned both strengths and weaknesses for the same interface, for example, appreciating the Autoplay ITUI’s passive operation while simultaneously criticizing its rigidity. This nuanced feedback highlights the importance of not only measuring overall preference but also of analyzing the balance of benefits and limitations perceived within each interaction model.

Beyond these primary themes, participants suggested additional improvements, such as integrating distinct audio tones for different types of system responses and enabling further customization of playback speed settings. These recommendations reinforce the strong link between interaction flexibility and user confidence. Moreover, numerous participants explicitly associated control features and feedback clarity with a heightened sense of independence. As one participant articulated, *“Being able to control the sound and know exactly what was happening made me feel I could explore without needing anyone to explain things to me.”*

While specific task completion times were not formally recorded, observational data and video analysis revealed clear trends in interaction fluency and need for assistance. Participants using the Pushbutton ITUI demonstrated longer sustained engagement without intervention and typically completed tasks independently after minimal orientation. In contrast, interactions with the RFID ITUI frequently required researcher guidance, particularly when participants struggled to locate the scanning zone or confirm successful activation. The Autoplay ITUI was generally easier to operate without help, but limited user agency due to its fixed playback structure. These patterns, although qualitative, support the quantitative findings regarding perceived control and independence.

Overall, the qualitative findings indicate that immediate, unambiguous feedback, fine-grained user control, and intuitive spatial design are key enablers of autonomous and satisfying museum exploration for BPS visitors. These user-driven insights offer practical guidance for the future development of ITUIs and highlight the critical role that physical interaction design plays in creating truly inclusive museum experiences.

## 5. Discussion

The findings from this study provide insights into how interactive technology can enable independent museum exploration for BPS visitors. The comparative analysis of three distinct interaction paradigms reveals critical factors that influence the effectiveness of accessibility solutions in cultural spaces and confirms our initial hypotheses. It is important to note that even though the issue of the importance of the audio commentary and audio control seem quite trivial, it seems that these aspects are somehow neglected/missed, and this is why this study focused on them.

### 5.1. Interaction Methods and User Independence

The Pushbutton ITUI consistently outperformed both the Autoplay and RFID systems across all measured metrics (usability, perceived independence, and user control). These results confirm our hypotheses and align with prior research suggesting that simple, tactile interfaces may outperform more technologically complex systems when they provide users with clear feedback and interaction flexibility (audio control and user control interface) [9,63,64].

The superior performance of the Pushbutton system aligns with key principles of universal design [65], which emphasize equitable, intuitive, and perceptible use. First, its equitable use is demonstrated through its accessibility to all users, regardless of vision level or prior technical experience. The physical layout and tactile buttons provide simple and intuitive use, reducing the cognitive demands of navigation. Immediate audio feedback satisfies the principle of perceptible information, allowing users to confirm actions non-visually. The button locations are consistent and distinguishable, demanding low physical effort and tolerating error, as incorrect inputs could be corrected easily without system failure. Finally, features such as volume and playback control offer flexibility, enabling visitors to tailor the experience to their processing pace and hearing needs. The strong correlation between control feature satisfaction and perceived independence (r = 0.78, *p* < 0.001) confirms H2, demonstrating that granular user control directly translates to enhanced autonomy. Specifically, volume adjustment (rated “very important” by 92% of participants) and playback speed control (rated “essential” by 88%) emerged as critical independence enablers. These design strengths reinforce the Pushbutton ITUI’s superiority in supporting independent exploration, functionally and ethically, by empowering users through inclusive design.

The limitations of the Autoplay ITUI’s operational model point to a fundamental tension in accessible design that warrants deeper theoretical examination. While hands-free operation might intuitively seem to lower access barriers, the findings suggest that restricting user control can compromise users’ sense of independence—a paradox that challenges traditional frameworks for conceptualizing accessibility. These conclusions align with the findings of Reichinger et al. [66], who emphasized the importance of tactile interaction and multimodal engagement for fostering autonomy, and with Vaz et al. [6], who highlighted the critical role of customizable audio feedback and tactile cues in supporting user independence.

Similarly, the challenges observed with the RFID scanning ITUI offer valuable lessons about the relationship between technological complexity and practical accessibility: increased technological sophistication does not necessarily translate into better user experiences if it introduces cognitive burden or operational uncertainty. This aligns with Pirrone et al.’s [7] assertion that many accessibility initiatives fail due to insufficient focus on user-centered design principles and the neglect of cognitive demands. Furthermore, Wang [8] emphasized that successful accessibility solutions must carefully balance innovation with usability to minimize cognitive load.

These insights suggest a pressing need to reconceptualize accessible design approaches in cultural spaces. Future research should systematically investigate how varying levels of user control impact both the practical aspects (e.g., ease of use, task completion) and the psychological dimensions (e.g., perceived autonomy, confidence) of independent exploration. As Story et al. [65] articulated in their universal design principles, effective accessibility solutions must prioritize user agency alongside physical interaction opportunities. Similarly, the Smithsonian Guidelines for Accessible Exhibition Design [45] advocated for a holistic approach that empowers users through intuitive, self-directed interaction rather than relying solely on physical access mechanisms or automated functionalities.

The analysis of the quantitative results reveals that independence in museum contexts encompasses multiple, interrelated dimensions: control over information flow, spatial navigation confidence through hand interaction, and the ability to manage the interaction experience autonomously.

### 5.2. Enhanced Audio Control Features

Participants emphasized volume adjustment (rated as “very important” by 92%) and playback speed control (rated as “essential” by 88%), demonstrating that fine-grained user control is not a peripheral preference but a core driver of independent engagement. While Avni et al. [9] reported moderate preference differences between interaction methods, our integration of playback speed control (0.5× to 2.0×), volume adjustment (±20 dB), and instant replay functionality resulted in large effect sizes (ηp^2^ = 0.39) and near-universal user approval (>88% rating as “essential”). For example, our participants repeatedly noted that being able to slow down playback allowed them to better understand spatial descriptions and engage more confidently with the replicas.

### 5.3. Beyond Physical Access: Toward Cognitive and Narrative Accessibility

Interactive systems that incorporate audio descriptions have become essential in shaping accessible museum experiences for BPS visitors. Central to this approach is balancing automated guidance with user autonomy—a principle emphasized in the recent literature. Leporini et al. [10] propose four key guidelines for effective interaction: consistent feedback, user-controlled pacing, multimodal delivery, and adaptable interfaces. These principles are reflected in Wang et al.’s [24] findings, which demonstrated increased engagement when users can control the pace and sequence of audio content. Hutchinson and Eardley [25] further showed that well-structured audio descriptions enhance both cognitive retention and emotional engagement for BPS and sighted audiences alike.

Expanding this framework, Wang [8] and Avni et al. [9] provided empirical support for the role of user control in shaping meaningful experiences. Wang [8] underscored the need for personalized descriptive systems that adapt to the user’s preferred pace and sequence. Similarly, Avni et al. [9] showed that systems offering greater physical control led to a stronger sense of independence. In our study, the Pushbutton interface, in particular, scored higher than the others because it enabled users to manage content delivery directly, highlighting that independence is rooted more in user agency than in technical complexity. This perspective complements Reichinger et al.’s [49] emphasis on multimodal systems by stressing that without meaningful control, even technologically advanced platforms may fail to support autonomous exploration.

While “control feature satisfaction” and “perceived independence” were strongly correlated in participants’ responses, they represent related but distinct constructs. Satisfaction with control features refers to the usability and responsiveness of specific interaction mechanisms, such as the ability to pause, repeat, or skip audio, while “independence” encompasses a broader sense of agency, including the ability to explore without assistance, initiate interaction, and self-pace learning. The high correlation suggests that users perceive responsive control as a key enabler of independence, but they are not conceptually identical. For instance, a participant might rate control features highly yet still feel dependent if they require spatial guidance or if the audio content is too vague. Future work could further separate these concepts using targeted questions or behavioral independence metrics.

These findings hold concrete implications for curators, designers, and accessibility consultants. Prioritizing adjustable, user-driven systems and embedding them within the exhibit narrative ensures that BPS visitors can engage with content meaningfully, confidently, and on their terms. Designing for independence, then, becomes a matter of designing for choice—both in content engagement and in physical navigation—while maintaining a coherent and accessible user experience throughout the space.

While the physical interface played a key role in enabling interaction, participants repeatedly stressed that the structure and clarity of the audio content were equally important. The organization, tone, and descriptive detail of the explanations affect their ability to understand and value the exhibits. Several participants mentioned that even a well-designed interface felt less effective when the audio was unclear, too fast, or lacked spatial orientation cues. This highlights that accessibility in museum experiences depends not only on hardware or interaction methods but also on content quality and pedagogical design. Future accessibility efforts should combine technological solutions with clear, layered, and user-focused interpretive content to ensure meaningful and independent engagement.

### 5.4. Limitations

As in any study, this too has several limitations that should be considered when interpreting its findings.

First, the sample size was relatively small, limiting the generalizability of the results. While the study provides valuable insights, a larger participant pool with diverse demographic and experiential backgrounds would strengthen the validity of the findings.

Second, the study was conducted in a specific setting, with a small, thematic set of exhibits on each ITUI prototype. While this provided a controlled environment to evaluate the prototypes, it may not fully reflect the challenges or opportunities of larger, more complex museum spaces or diverse exhibit types, or bigger kits.

Third, the ITUIs were designed with specific interaction paradigms (Autoplay, Pushbutton, and RFID scanning) in mind, which, while innovative, may not cover the full range of possible interaction methods for BPS visitors. Other approaches, such as voice-activated systems, adaptive AI-driven interfaces, or multisensory feedback mechanisms, should be explored in future research to broaden the scope of accessibility solutions.

Fourth, the independence metrics used in this study, while valuable, are inherently subjective and susceptible to potential biases. Factors such as participants’ familiarity with similar technologies, personal preferences for tactile interfaces, and environmental conditions during testing (e.g., background noise) might have influenced the results.

Lastly, while the study emphasizes independence, it does not fully explore the social dimension of museum visits, such as how ITUIs might support collaborative exploration with companions or enhance group experiences. Investigating how these technologies can facilitate both individual and social engagement could provide additional insights for designing inclusive museum environments.

## 6. Conclusions and Future Work

While recent technological innovations have begun to bridge this gap, many accessibility solutions focus primarily on basic inclusion rather than fostering independent exploration. This research addresses this limitation by developing features that empower visitors’ autonomy through customizable interaction patterns and self-paced exploration, specifically, by providing audio control features. Collectively, the evidence points to a multifactorial foundation for independence, which arises from the interplay of physical control (e.g., tactile input), cognitive confidence (autonomy and ease-of-use ratings), and customization capabilities. Beyond the interface itself, the research highlights that true accessibility requires integration into the overall museum design. Participants’ feedback consistently pointed to the importance of spatial organization, intuitive layout, and the seamless incorporation of interactive elements. As such, accessibility should not be viewed as an add-on but rather as a guiding framework in exhibition planning. Effective solutions will embed interaction points naturally within exhibit flows, use clear physical and semantic cues, and provide multi-sensory anchors that support spatial orientation and engagement.

Practically, when designing an ITUI to be used by BPS visitors, one should ensure to provide a clear and detailed description of the ITUI and how to operate it from the point of view of BPS visitors. Another aspect is that the audio commentary should closely refer to the ITUI itself, helping the visitor to understand it by touch. Finally, the ability to control the audio, volume, speed, stopping/replaying, etc., needs careful attention.

While this study makes a useful contribution by refining interactive audio–tactile interfaces for blind museum visitors, its proposed enhancements, such as improved audio structure and playback control, represent a small step within a well-established design space. Future work could explore more transformative approaches to accessibility. For example, integrating AI-driven conversational agents (e.g., ChatGPT) to provide dynamic, user-driven descriptions could significantly improve interaction. These agents would enable users to ask context-specific questions about the artifacts, encouraging active inquiry rather than passive reception. Even a Wizard-of-Oz prototype could simulate such interactions in early stages. Additionally, future studies might evaluate conceptual understanding or retention after interaction (e.g., through comprehension questions), allowing for a deeper assessment of educational value alongside usability.

Moreover, future research should recruit participants with varying levels of visual impairment, familiarity with assistive technologies, and cultural or geographic contexts to ensure broader applicability. It should also explore the scalability and adaptability of the ITUIs in a variety of exhibition formats, including large-scale museums.

## Figures and Tables

**Table 1:** Usability, independence, and control metrics across ITUIs. * Indicates statistical significance at *p* < 0.05.

Measure	Pushbutton ITUI	Autoplay ITUI	RFID ITUI	Test Statistic	*p*-Value
SUS score mean (SD)	87.5 (±8.2)	76.3 (±9.4)	68.9 (±11.2)	F = 12.34	<0.001 *
Independent operation	18 (72%)	5 (20%)	2 (8%)	Χ^2^ = 19.28	<0.001 *
Sense of control	19 (76%)	4 (16%)	2 (8%)	Χ^2^ = 21.44	<0.001 *

**Table 2:** Mean of user satisfaction ratings by interface type. Tukey’s post hoc results for pairwise comparisons are summarized in-text.

Dimension	Pushbutton	Autoplay	RFID	F(2,72)	*p*-Value
Physical characteristics	4.8 (±0.2)	4.5 (±0.3)	4.2 (±0.4)	9.76	<0.001 *
Usability	4.7 (±0.3)	4.2(±0.4)	4.1 (±0.5)	11.23	<0.001 *
Safety	4.6 (±0.3)	4.7 (±0.2)	4.3 (±0.4)	5.87	<0.004 *
Effectiveness	4.8 (±0.2)	4.4 (±0.3)	4.2 (±0.4)	10.45	<0.001 *
Control features	4.7 (±0.3)	4.0 (±0.5)	4.1 (±0.4)	12.78	<0.001 *

* Indicates statistical significance at *p* < 0.05.

## Data Availability

The original contributions presented in this study are included in the article material. Further inquiries can be directed to the corresponding authors.

## Abbreviations

The following abbreviations are used in this manuscript:

BPS	Blind and Partially Sighted
ITUI	Interactive Tangible User Interface
SUS	System Usability Scale
RFID	Radio-Frequency Identification
AUSQ	Additional User Satisfaction Questions
TPQ	Two-Part Questionnaire
QUEST	Quebec User Evaluation of Satisfaction with Assistive Technology
IRB	Institutional Review Board
SPSS	Statistical Package for the Social Sciences
ANOVA	Analysis of Variance
HSD	Honestly Significant Difference (Tukey’s HSD)
STL	Stereolithography (3D printing file format)
WHO	World Health Organization

## Appendix A. Demographics

### Appendix A.1. Demographics and Museum Visit Preferences

**Table A1:** Demographics and museum visit preferences.

ID	Age	Gender	Years of BPS	Visit Museums?	Frequency	Accompanied by	BPS/Not Accompany	Guided Tour	Family or Friend	Auditory Guide	No Assistant	Touch Screens	App	NFC Tech. Familiarity
1	58	F	58	yes	few times a year	couple	no preference	V	V	V		V	V	Yes
2	44	F	15	no					V			V		Yes
3	34	F	34	no				V				V	V	No
4	27	F	27	no				V	V	V		V		Yes
5	23	F	23	no				V	V	V			V	Yes
6	26	F	26	no					V	V			V	Yes
7	21	F	21	yes	once every few years	family or friends	no preference		V		V	V		Yes
8	21	M	21	no				V						Yes
9	21	F	21	yes	once every few years	family or friends	not blind partner	V	V	V		V		Yes
10	25	F	25	no				V	V	V		V	V	Yes
11	19	M	19	no				V	V			V	V	Yes
12	23	M	23	yes	once every few years	family or friends	not blind partner	V	V					Yes
13	29	M	29	no				V	V			V	V	Yes
14	49	F	49	yes	once v few years	family or friends	blind partner	V						Yes
15	23	M	23	yes	once v few years	family or friends	not blind partner	V	V					Yes
16	21	M	21	no				V	V					No
17	21	M	21	yes	once a year	family or friends	blind partner	V	V					Yes
18	21	F	13	no				V	V	V		V		Yes
19	48	F	30	yes	once a year	family or friends	not blind partner	V	V			V		Yes
20	50	F	32	no					V					Yes
21	23	F	23	no				V						Yes
22	32	F	26	no				V				V		No
23	36	M	36	yes	once every few years	family or friends		V	V	V		V	V	No
24	31	M	31	yes	once a year	family or friends	no preference	V	V	V			V	Yes
25	30	F	30	yes	once every few years	family or friends	not blind partner	V	V	V			V	Yes

### Appendix A.2. Education and Profession Demographics

**Table A2:** Education and profession demographics.

Education Level *	Number of Participants	Common Professions
Primary or lower	3	Psychometric exam preparation, programming courses, unemployed
Secondary/tertiary (BA)	17	Social workers, engineers, teachers, instructors, business owners
Graduate and above (MA)	5	Special education teachers, project coordinators, graduate students

* Education categories based on WHO-style population standards.

## Appendix B. Research Questionnaires

### Appendix B.1. System-Specific Satisfaction and Usability Questionnaire

Each item was rated on a five-point Likert scale (1 = strongly disagree, 5 = strongly agree)

1. I do not think I would want to use this ITUI often.
2. I think the ITUI is unnecessarily complex.
3. I found the ITUI easy to use.
4. I think I would need help to use the kit.
5. I think the different functions of the ITUI are well integrated.
6. I encountered many inconsistencies throughout the use of the kit.
7. I think most people can learn to use the ITUI quickly.
8. I think the ITUI is cumbersome to use.
9. I felt very confident in how I used the kit.
10. I had to learn a lot before I was able to use the kit.
11. I am satisfied with the kit’s dimensions (size, height, length, width) and exhibits.
12. I am satisfied with the weight of the exhibits.
13. I am satisfied with the ease with which the exhibits can be removed and returned to their place.
14. I am satisfied with how safe and secure the accessory is.
15. I am satisfied with the durability of the exhibits (that they will not break in your hand).
16. I am satisfied with the easy use of the ITUI (starting and stopping the audio files).
17. I am satisfied with how comfortable the ITUI is.
18. I am satisfied with the kit’s effectiveness (how well this device fulfills your needs).

### Appendix B.2. Comparative Assessment Questionnaire

1. Overall, which ITUI was easier to operate?
2. Overall, which ITUI was more convenient to operate?
3. Overall, which ITUI was more efficient to operate?
4. Overall, which ITUI was more independent to operate?
5. Which ITUI gave you a sense of control over the situation?
6. Which ITUI was easy to learn?
7. Overall, which ITUI do you prefer?

### Appendix B.3. General Preferences Questionnaire

Each item was rated on a seven-point Likert scale (1 = strongly disagree, 7 = strongly agree)

1. I generally prefer products and devices with buttons that can be pressed.
2. I generally prefer methods of interaction with the ITUI using RFID technology.
3. I prefer to listen to explanations in the museum with headphones.
4. I prefer to listen to explanations in the museum with speakers that those visiting with me can also hear.
5. The ability to change the reading speed of the audio system is important.
6. The ability to control the volume is important.
7. To the extent that the audio file can be started, there should be an option to stop the recording.
8. I would prefer a similar application that is installed on my phone.
9. The ability to touch and hold the printed exhibits enhances understanding and contributes to the experience.
10. The ability to hear the audio descriptions contributed to a high level of independence in understanding the cultural and historical contexts of the exhibits in the ITUIs.
11. Kits of this type can enrich and upgrade the experience of visiting the museum independently for me.

## Appendix C. Qualitative Feedback Analysis

### Appendix C.1. Participant (P) Comments by Theme

#### Appendix C.1.1. Feedback Clarity

##### Pushbutton ITUI

- P7: The immediate click response let me know exactly what I was doing. Each button press gave clear feedback, which made me confident in my interactions.
- P15: I always knew when I had successfully activated a feature. The tactile feedback was consistent and reassuring.
- P22: The mechanical feedback from the buttons was perfect—not too hard to press, but clear enough to know when you have pressed it.
- P4: Unlike other ITUIs I have used, there was never any doubt whether I had activated something.
- P18: The combination of the click feeling and the immediate audio response made navigation very straightforward.

##### Autoplay ITUI

- P3: Sometimes I was unsure if I had placed the object correctly to trigger the audio.
- P11: The automatic activation was nice, but I wished there was clearer feedback when the ITUI recognized the object.
- P25: It worked well when it worked, but sometimes I was uncertain if I had positioned things correctly.

##### RFID ITUI

- P9: The scanning feedback was inconsistent—sometimes I could not tell if I was holding the tag in the right place.
- P13: I spent too much time finding the correct scanning position.
- P20: The lack of immediate feedback made it difficult to know if I was using it correctly.

#### Appendix C.1.2. Control Granularity

##### Pushbutton ITUI

- P3: I could easily control the pace of information, which helped me absorb complex details.
- P12: The ability to repeat sections was crucial for understanding. I could focus on specific details without losing my place.
- P8: Having separate buttons for different functions made it easy to navigate between sections.
- P17: I appreciated being able to adjust the speed and volume independently.
- P24: The control layout made sense—everything was where I expected it to be.

##### Autoplay ITUI

- P6: I wished I had more control over the playback without removing and replacing the object.
- P14: The automatic playing was convenient but inflexible. Sometimes, I needed to hear something again.
- P21: It would be better to pause or restart without manipulating the object.

##### RFID ITUI

- P2: The scanning concept was interesting but needed more precise control options.
- P16: I found it difficult to scan specific sections when I wanted to review information.
- P23: “The ITUI could benefit from more granular control over the audio playback”.

#### Appendix C.1.3. Spatial Organization

##### Pushbutton ITUI

- P5: The systematic arrangement of buttons helped me create a mental map of the exhibit.
- P10: I quickly learned where everything was–the layout was very logical.
- P19: The consistent spacing between controls made navigation intuitive.
- P1: The symmetrical arrangement helped me remember where different functions were located.
- P25: Even without seeing the layout, I could understand how the controls were organized.

##### Autoplay ITUI

- P7: The fixed positions of objects helped with orientation, but the lack of interactive elements made it less engaging.
- P15: I sometimes lost track of where I was in the exhibit space.
- P22: The spatial relationship between objects was unclear.

##### RFID ITUI

- P4: Finding the correct scanning location was challenging without clear physical markers.
- P11: The scanning zones were not intuitive to locate.
- P18: I needed more tactile cues to understand the spatial layout.

### Appendix C.2. Additional Observations and Suggestions

#### Appendix C.2.1. ITUI Improvements

- P8: Adding audio confirmation of speed and volume changes would be helpful.
- P13: Some form of tactile marking to indicate optimal scanning positions would improve the RFID ITUI.
- P16: A way to bookmark or flag specific sections for later review would be useful.
- P20: The ability to customize button sensitivity would help users with different motor control abilities.

#### Appendix C.2.2. General Comments on Independence

- P2: The Pushbutton ITUI gave me the most confidence to explore independently.
- P9: Being able to control the pace and review content made me feel more autonomous.
- P17: Clear feedback was essential for independent navigation and learning.
- P23: The more control I had over the interaction, the more comfortable I felt exploring on my own.

## Appendix D. SUS Scores by Item and ITUI

**Table A3:** SUS scores by item and ITUI.

Item	RFID			AutoPlay			Pushbutton		
	M	SD	Median	M	SD	Median	M	SD	Median
1. I think that I would like to use this system frequently.	4.48	0.82	5.00	4.12	1.17	5.00	4.52	0.77	5.00
2. I found the system unnecessarily complex.	1.44	0.65	1.00	1.40	0.87	1.00	1.16	0.67	1.00
3. I thought the system was easy to use.	4.72	0.54	5.00	4.48	0.87	5.00	4.84	0.37	5.00
4. I think that I would need the support of a technical person to be able to use this system.	1.76	0.93	1.00	1.96	1.21	1.00	1.12	.033	1.00
5. I found the various functions in this system were well-integrated.	4.84	0.37	5.00	4.68	0.90	5.00	4.92	0.28	5.00
6. I thought there was too much inconsistency in this system.	1.48	0.77	1.00	1.64	1.22	1.00	1.24	0.72	1.00
7. I would imagine that most people would learn to use this system very quickly.	4.72	0.54	5.00	4.56	0.71	5.00	4.88	0.33	5.00
8. I found the system very cumbersome to use.	1.12	0.33	1.00	1.28	0.79	1.00	1.08	0.28	1.00
9. I felt very confident using the system.	4.60	0.82	5.00	4.32	0.90	5.00	4.76	0.83	5.00
10. I needed to learn many things before I could get going with this system.	1.28	0.79	1.00	1.77	1.04	1.00	1.12	0.33	1.00

SUS score—means, standard deviations, and medians of transformed and reversed (0–4 scale).
